# Quality and Content of Bioactive Compounds in Muffins with Residue After Isolation of Starch from Unripe Apples (*Malus domestica* Borkh)

**DOI:** 10.3390/molecules30102189

**Published:** 2025-05-16

**Authors:** Dorota Gumul, Stanisław Kowalski, Anna Mikulec

**Affiliations:** 1 Department of Carbohydrate Technology and Cereal Processing, Faculty of Food Technology, University of Agriculture in Krakow, 122 Balicka Street, 30-149 Krakow, Poland; st.kowalski@urk.edu.pl; 2 Faculty of Engineering Sciences, University of Applied Science in Nowy Sacz, 33-300 Nowy Sacz, Poland; amikulec@ans-ns.edu.pl

**Keywords:** by-products, unripe apples, functional foods, circular economy

## Abstract

Growing consumer awareness encourages food producers to look for new fortifying additives for muffins. One such additive may be the polysaccharide fraction residue after starch isolation from unripe apples, as they are a source of many bioactive compounds. The aim of the study was to examine the effect of the addition of the polysaccharide fraction residue from unripe apples on the quality and physical properties as well as health-promoting properties of muffins. It was observed that the polysaccharide fraction residue from unripe apples did not deteriorate the texture or volume of muffins and contributed to an increase in the content of polyphenols, and the antioxidant potential of muffins, especially the polysaccharide residue from unripe apples of the Oliwka variety, had a more beneficial effect on the above-mentioned features of muffin than Pyros. Moreover, it was observed that the content of phytosterols (campesterol and cleosterol) in muffins increases but the content of tocopherols decreases due to their thermolability during the baking process.

## 1. Introduction

Apples (*Malus domestica* Borkh) are an extremely popular fruit in many countries located in different geographical zones and their production is increasing year by year (in the last decade, it has increased by 38%, i.e., from 55 million tons to 81 million tons). These fruits are preferred by consumers due to the fact that they contain a number of nutrients (sugars, macro and microelements—especially K, P, Mg, Ca, protein) and health-promoting compounds (such as vitamin C, flavonoids and phenol compounds, phytosterols, dietary fiber—pectin, cellulose, hemicellulose, and lignin, fatty acids—linoleic, oleic, palmitic acid, and organic acids and also carotenoids) [1,2,3,4,5].

Apples are mainly used for the production of juice, wine, cider, and fruit purees but also for direct consumption [6,7].

Unfortunately, the production of the products presented above is associated with the generation of a very large amount of apple waste, which includes seeds, stalks, pulp with skin, husk, and seed nests, which are commonly called apple pomace. This diversity of morphological parts included in the microbiologically stable pomace after drying makes it a potential source of many valuable compounds such as proteins, minerals and vitamins, and primarily dietary fiber and phytochemicals including polyphenols, organic acids, aldehydes, alcohols, color, and aromatic substances [8,9].

The reuse of the above-mentioned compounds is justified and fits perfectly into the current trend of zero waste technology. Zero waste technology assumptions and practices are strongly linked to several of the 17 UN Sustainable Development Goals—SDGs [10]—especially with goal no. 12 concerning the need to reduce waste, increase recycling and promote sustainable production practices in the industry, especially the food industry. Post-production raw materials that are a heterogeneous mixture of morphological parts of the plant constituting a specific nutritional matrix could be a valuable source of health-promoting compounds, not only dietary fiber but above all polyphenolic compounds with chemopreventive effects (anti-carcinogenic, reducing postprandial glucose levels and arterial hypertension, anti-inflammatory properties, antiviral, antibacterial, antiallergic and anticoagulant properties, and can reduce the risk of occurrence of diseases such as atherosclerosis and other cardiovascular diseases, and neurodegenerative diseases including Alzheimer’s and Parkinson’s disease) [11,12,13,14,15].

Therefore, they should be used to produce innovative food products; however, for many years, waste raw materials (solid or liquid) were rarely used in the production chain and were often used to produce animal feed, fertilizers or, like apple peel, to produce pectin. Unfortunately, a large part of this waste remained unprocessed, which caused additional disposal costs and increased the biological load of sewage [16,17].

That is why it is so important to use apple pomace because it is a valuable source of not only polyphenols (chlorogenic acid, catechin, epicatechin, quercetin derivatives, procyanidin B2, and phlorizin, the latter being unique) [9,18,19] but also dietary fiber, which has hypoglycemic and hypocholesterolemic properties and reduces the risk of atherosclerosis, coronary heart diseases, large intestine cancer, and diabetes by decreasing blood glucose levels [20]. The reduction in blood glucose levels is, apart from the presence of fiber, mainly related to the presence of a polyphenol from the dichydrochalcone group in apple pomace—phlorizin, which is an inhibitor of glucose “uptake” in the small intestine and kidneys. This compound is a competitive inhibitor of the glucose transporter combined with sodium in the small intestine and the apical region of the renal proximal tubule. Due to limited oral bioavailability, the isolated form of phlorizin cannot be used as a medicine, but phlorizin in apple pomace, which is a nutritional matrix, still maintains a therapeutic effect [21].

Due to its relatively low cost and potential nutritional and pro health value, apple pomace has been considered as an attractive functional ingredient to formulate functional foods. Apple pomace is very often incorporated into cookies because they are widely eaten by consumers of all ages around the world due to their affordability, long shelf life, and high nutritional value [22]. Cookies can be a suitable matrix for incorporating various substances rich in bioactive compounds [23]. Currently, some studies focus on the use of apple pomace in cakes and other sweet bakery products to improve their sensory properties and nutritional composition and dietary and health-promoting value [2,24,25,26,27,28].

Another possibility of managing the apple production was presented in the earlier studies by Gumul et al. [29], which concerned obtaining starch with very valuable functional properties derived from unripe apples. The residue that accompanies this process may contain large amounts of polyphenols because, according to the literature [30,31], unripe apples are characterized by a very large amount of polyphenols, especially phenolic acids, ten times greater than in ripe apples. Therefore, the study on this residue and the respective muffins is deeply justified. This approach should also be considered within the broader context of climate change. This option should also be paid attention to in the context of hailstorms during the ripening of apple fruit. Unripe apples harvested on this occasion could be used for isolation of starch with a low tendency to retrogradation. Such type of starch is extremely valuable because starch retrogradation is one of the main factors causing the deterioration of food quality and among others may be largely responsible for the staling of baked foods [32]. The polysaccharide fraction residue after isolation of starch may have a large health-promoting potential due to the previously mentioned significant amount of phenolic compounds, which can be successfully utilized in food technology. This approach is innovative in relation to research on the utilization of ripe apple pomace for enriching cookies, which has been popular in recent years [2,24,25,26,27,28]. Therefore, the aim of this study was to investigate the effect of the polysaccharide fraction residue from different varieties of unripe apples (Oliwka and Pyros) on the amount of health-promoting compounds from the groups of polyphenols, tocopherols, and phytosterols in wheat muffins. Moreover, the antioxidant potential of muffins with the addition of the polysaccharide fraction from two varieties of unripe apples was estimated. Further, the physical characteristics (volume, texture) of wheat muffins with the addition of the polysaccharide fraction were determined.

## 2. Results and Discussion

### 2.1. Characterization of the Polysaccharide Fraction Residue Resulting from the Isolation of Starch from Unripe Apple Varieties—Oliwka and Pyros

**Figure 1:**
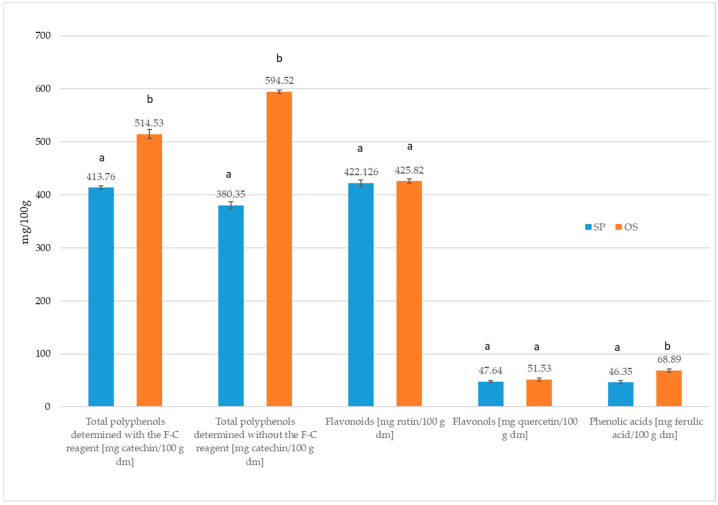
The content of polyphenols and flavonoids, phenolic acids, and flavonols in the analyzed polysaccharide fraction residue after isolation of starch from Pyros and Oliwka unripe apples. Total polyphenols were determined with the F-C reagent [mg catechin/100 g d.m.]; total polyphenols were determined without the F-C reagent [mg catechin/100 g d.m.]; flavonoids [mg rutin/100 g d.m.]; flavonols [mg quercetin/100 g d.m.]; phenolic acids [mg ferulic acid/100 g d.m.]. Different letters indicate statistically significant differences (Duncan’s test, significance level α ≤ 0.05).

In the first stage of the work, bioactive compounds, i.e., polyphenols, tocopherols, and phytosterols, were characterized and the antiradical and antioxidant activity of the polysaccharide fraction residue was determined. Figure 1 presents the total phenolic content determined using the Folin–Ciocalteau reagent (F-C reagent) (as well as without this reagent), along with the flavonoids, phenolic acids, and flavonols in the polysaccharide fraction residue after the isolation of starch from unripe apples varieties Pyros and Oliwka. It was found that the polysaccharide fraction residue from unripe apple variety Oliwka (SO) contained 24% more polyphenols than the polysaccharide fraction residue from unripe apple variety Pyros (SP). On the other hand, in the case of flavonoid content, no differences were noted between these samples (*p* < 0.05).

It was found that the total phenolic content (TPC), without the Folin–Ciocalteau reagent in SO, was 56% higher than the content of polyphenols in the SP. SO was characterized by a higher content of phenolic acids by 49%, compared to SP, but the content of flavonols was at the same level *p* < 0.05.

In order to compare our research with the research of other authors, TPC was converted to gallic acid equivalents. It was obtained that in SO, total phenolic content was at the level of 257.14 mg gallic acid/100 g d.m, and in SP, 208.28 mg gallic acid/100 g d.m. In the study of Candrawinata et al. [33], concerning apple pomace from ripe apples, the total phenolic content was 118.6 mg gallic acid/100 g d.m, while Adil et al. [34] determined the total polyphenol content at the level of 47 mg gallic acid/100 g d.m. On the other hand, Bai et al. [35] reported a value of 62.7 mg gallic acid/100 g d.m. in apple pomace. According to Persic et al. [36], Gumul et al. [37], and Muresan et al. [38], the total polyphenolic content in apple pomace was in the range 19–89 mg gallic acid/100 g d.m. By comparing the total phenolic content in polysaccharide fraction residue from unripe apples (approximately 232.5 mg gallic acid/100 g d.m.) considered in this work with the results of the above-mentioned authors concerning TPC in apple pomace from ripe apples, it was clearly established that the content of polyphenols in polysaccharide fraction residue from unripe apple is 2 to 11 times higher (Figure 1) compared to the content of these compounds in apple pomace from ripe apples [33,34,35,36,37,38]. In this context, it can be stated that polysaccharide fraction residue obtained from unripe apples is a rich source of polyphenol content compared to apple pomace from ripe apples. Also, the flavonoid content was about four times higher in polysaccharide fraction residue obtained from unripe apples (about 410 mg rutin/100 g d.m.) than in apple pomace from ripe apples. The total content of flavonoids in apple pomace from ripe apple was in the range from 94.3 mg rutin/100 g d.m. [37] to 119 mg rutin/100 g [39].

**Figure 2:**
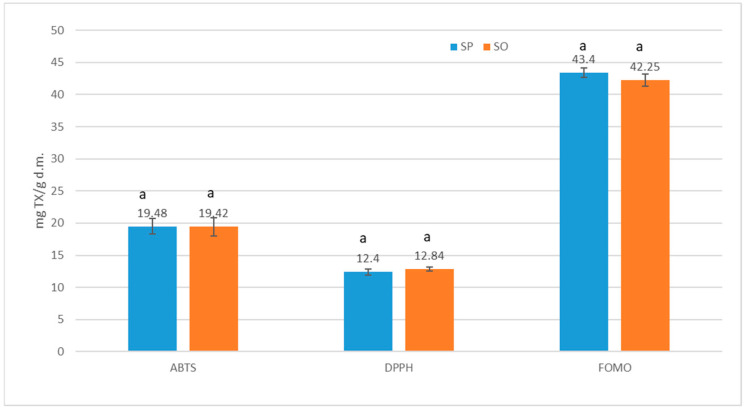
Antiradical and antioxidant activities in the analyzed polysaccharide fraction residue after isolation of starch from Pyros and Oliwka unripe apples. The same letters indicate no statistically significant differences (Duncan’s test, significance level α ≤ 0.05).

Figure 2 presents the antiradical and antioxidant activities of the analyzed polysaccharide fraction residue after isolation of starch from unripe apples. The same antiradical and antioxidant activities were noted, marked with the free radical ABTS, DPPH, and FOMO, regardless of the polysaccharide fraction residue originating from two different apple varieties, although the content of polyphenols, phenolic acids, and flavonols was higher in SO than SP. Therefore, it is believed that substances other than polyphenols, such as vitamin E, vitamin C, glutathione, enzymes, and polyphenolic substances, which can be transformed into more reactive compounds even during the isolation of these polysaccharides fraction residue from apples, are also involved in the antioxidant activities [40]. Comparing the results of the study by Gumul et al. [37] on apple pomace with the results of this work (Figure 2), it was proven that the antiradical activity determined by the ABTS method was twice as high in polysaccharide fraction residue from unripe apples compared to apple pomace [37].

In addition, it was found that a greater amount of alpha and gamma tocopherol was present in SP than SO, in contrast to delta-tocopherol. The content of alpha tocopherol was twice as high in SP than SO, and the content of gamma tocopherol was 12% higher in SP than SO. The content of delta-tocopherol was 89% higher in SO than SP (Table 1).

Cholesterol and stigma-sterol in both polysaccharide fraction residue were identical (*p* < 0.05), while the content of the remaining ones was different. The content of campesterol was greater by about 11% in SO than SP (Table 1).

The content of beta-sitosterol was 14% higher in SP than SO, in contrast to cleosterol, the content of which was lower in SP than SO. It should also be noted that the dominant phytosterol in the residue after starch extraction from unripe apples was beta-sitosterol, followed by campesterol (Table 1). Also Woźniak [41] and Radenkovs [42] indicated that apple pomace is very rich in beta-sitosterol and campesterol. Due to the fact that phytosterols are structural and functional analogs of cholesterol synthesized by plants, these compounds are part of the composition of plant cell membranes, hence their large amount in the polysaccharide fraction residue after starch isolation from unripe apples. The dominant apple phytosterol is beta-sitosterol with campesterol taking second place. In addition, Radenkovs [42] stated that apple pomace contains quite a large amount of alpha tocopherol as well as beta and gamma tocopherol.

It can therefore be said that the polysaccharide fraction residue from unripe apples is a source of many bioactive compounds, among which one should distinguish polyphenols, i.e., especially flavonoids, phenolic acids, and flavonoids. The above-mentioned polysaccharide fraction residues have quite high antiradical and antioxidant activity. In addition, these residues, after the isolation of starch from unripe apples, also have a high content of alpha tocopherol, gamma-tocopherol, and delta tocopherol and phytosterols.

The amount of polyphenols and other biologically active compounds from the groups of phytosterols and tocopherols was many times higher in polysaccharide fraction residue formed after the isolation of starch from unripe apples than in pomace from ripe apples. Therefore, we can consider polysaccharide fraction residue to be a very valuable, innovative, and functional food additive.

So far, no one has used such an additive to enrich cookies. Previous publications have focused on the possibility of enriching cookies with apple pomace from ripe apples [2,24,25,26,27,28]. Hence, considering such a possibility is a very innovative approach to ensure circular economy in the era of global climate change.

### 2.2. The Influence of Polysaccharide Fraction Residue Derived from Unripe Apple Oliwka and Pyros Varieties on the Polyphenol Contents in Muffins

Table 2 shows the total phenolic content in muffins with polysaccharide fraction residue. It was shown that the addition of polysaccharide fraction residue from unripe apples guaranteed an increase in the total phenolic content in muffins compared to the control (Table 2). In muffins with 5% SP, the total phenolic content was 35% higher compared to the control. An increase in the share of polysaccharide fraction residue of the same apple variety to 10% increased the total phenolic content by 37% compared to the control sample. On the other hand, 5% SO increased the total phenolic content in muffins by 42% compared to the control. The addition of 10% SO contributed to an increase in the total phenolic content in muffins by 84% compared to the control (Table 2). When comparing the polysaccharide fraction residue additives from two unripe apple varieties, it was clearly shown that SO was a much better additive than SP for baking muffins. This results from the fact that SO has a higher total phenolic content than SP (Table 2, Figure 1). A similar relationship was noted when the total phenolic content was determined without the F-C reagent. Muffins with SO were characterized by a higher total phenolic content ranging from 34 to 81% compared to the control. On the other hand, muffins with SP showed a higher content of polyphenols from 15 to 26% compared to the control (Table 2). In the case of flavonoids, the use of polysaccharide fraction residue from various apple varieties for baking muffins contributed to an increase in the content of these compounds in the muffins compared to the control. Muffins with a 5% share of SP were characterized by a higher content of flavonoids compared to the control by 78%. The remaining shares of 10% SP and 5 and 10% SO guaranteed an increase in flavonoids in muffins at the same level (about 108%) compared to the control. Due to the identical flavonoid content in the residues themselves, they should contribute to the identical increase in flavonoids in the muffins (Table 2, Figure 1). It was found that the content of phenolic acids increased by three to five times in muffins with polysaccharide fraction residue from unripe apples of both varieties compared to the control muffins. It was also observed that the increase in phenolic acid content in muffins with the above-mentioned additive was greater than that resulting from the level of the additive. This may be partly due to the thermal decomposition of quercetin derivatives, especially quercetin rutinoside, which generates phenolic acids [28,43,44].

The use of polysaccharide fraction residue of different apple varieties increased the content of flavonols in the finished muffins as relative to the control. Muffins with a 5% SP contained twice as much flavonols as the control. A 10% addition of SP guarantees a 3.6-fold increase in content of flavonols compared to the control. A similar increase in flavonols was observed in the case of the use of SO in muffins (Table 2).

Analogous results of the increase in the amount of polyphenols in muffins with various enriching additives were obtained by other authors. Ajila et al. [45], who added powdered mango peel in the amount of 20% in relation to the added wheat flour in cookies, noted a polyphenol content 7 times higher compared to the control [45]. A 6% addition of powdered apple peel increased the content of polyphenols and flavonoids in muffins by 226% and 45%, respectively, compared to the control sample [46]. In the study by Acun and Gul [47], 5% and 10% of grape pomace additives were used to bake muffins, which increased the polyphenol content in the cookies by five to six times, respectively, compared to the control. Cookies in which powdered tangerine peel was used as an additive in the amount of 6% of the flour used in the recipe caused a 2-fold increase in the polyphenol content compared to the control sample [48]. In the work of Caponio et al. [49], the polyphenol content in muffins with the addition of 10% and 20% of flour from wine lees was 2 and 5 times higher, respectively, compared to the control sample.

In summary, it should be noted that the baking process (temperature around 200 °C) causes the loss of some phenolic compounds by up to 60% [50]. These losses are influenced by many factors, such as thermal, enzymatic, oxidative degradation, as well as isomerization/epimerization processes, especially catechins [51] and decarboxylation of phenolic acids [50,52,53]. Additionally, losses of these phenolic compounds may be caused by the formation of complexes with polysaccharides [51,54]. Although thermal processes such as baking contribute to the loss of polyphenols, the polysaccharide fraction residue introduced into muffins, which are a powerful source of bioactive compounds, guarantees a significant content of phenolic compounds in these final products.

### 2.3. The Influence of Polysaccharide Fraction Residue Derived from Unripe Apple Oliwka and Pyros Varieties on the Antioxidant Capacity of Muffins

An increase in the antiradical activity determined by the synthetic ABTS radical was noted in muffins with the addition of SP and SO (Table 3). In muffins with 5% and 10% SP, the antiradical activity was higher by 28% and 45%, respectively, compared to the control. On the other hand, a 5% addition of SO incorporated into the muffin increased the antioxidant activity by 25% compared to the control, and a 10% addition of polysaccharide fraction residue from the same apple variety into the muffins by 53% compared to the control (Table 3). In the case of DPPH, it was observed that the antiradical activity of all muffins with polysaccharide fraction residue was identical to the control muffin. The exception in this respect was muffins with 5% SO, where the increase in antiradical activity towards the DPPH radical was within the range of 11% compared to the control. The results of this analysis are divergent in relation to the other analyses of antiradical and antioxidant activity of muffins with polysaccharide fraction residue. This may be explained by the fact that there was the interference of other compounds, in this case, carotenoids, which are absorbed at the same wavelength used in this determination, i.e., 517 nm [55]. The amount of carotenoids in unripe apples, especially the skin of these apples, is very large [56]; therefore, there was interference of polyphenols and carotenoids, hence the results of the DPPH determination are not very legible (Table 3).

The antioxidant capacity test (FOMO) showed significant differences between control muffins and those enriched with the addition of polysaccharide fraction residue from both unripe apples (Table 3). The control muffins had the lowest antioxidant activity, which increased with the addition of polysaccharide fraction residue, which was equivalent to an increase in antiradical activity. A 5% addition of SP (Table 3) caused an increase of the antioxidant activity of muffins by almost 6 times on the other hand with 10% addition by almost 10 times, while the addition of SO increased the activity in muffins by 8 to 14 times, respectively, for the same levels. Muffins with the addition of SO showed higher antioxidant activity than those with SP, although in the case of antiradical activity, no such relationship was noted (Table 3).

The difference in the results of DPPH and ABTS antiradical activity and FOMO antioxidant activity in the analyzed muffins should be explained by the fact that these methods are based on different principles and mechanisms taking into account the multidirectional action of polyphenols as antioxidants [57,58]. Also, each of these methods can act selectively on specific groups of antioxidants. Some researchers [59,60] suggest that the DPPH method may be more important in determining the activity of vitamin C (not determined in our study), while the other methods are used to determine mainly the content of polyphenols.

A strong correlation was observed between the bioactive compounds present in muffins after the introduction of polysaccharide fraction residues and the antioxidant potential of the muffins. Strong correlations were found between TPC and ABTS (r^2^ = 0.882) and between TPC and FOMO (r^2^ = 0.929). Similar significant correlations were observed between total flavonoids and FOMO (r^2^ = 0.937) and total flavonoids and ABTS (r^2^ = 0.917). Phenolic acid content correlated with ABTS (r^2^ = 0.911) and FOMO (r^2^ = 0.962). These strong correlations confirm the effectiveness of additives in improving the antioxidant properties of muffins. It should be emphasized that the antioxidant and antiradical activity may also be influenced by newly created substances with antioxidant properties during baking, i.e., Maillard reaction products, and transformations of phenolic compounds from a less to a more active form, as well as tocopherols and phytosterols present in the analyzed products [61,62,63]. Nevertheless, the above-mentioned strong correlation between antiradical and antioxidant activity and the content of polyphenols, flavonoids, and phenolic acids in muffins prove that these bioactive compounds mainly determine the high antioxidant potential of muffins (Figure S2).

In the work of Caponio et al. [49], the antioxidant activity measured against ABTS in muffins with a 10% addition of flour from wine lees was 2 times higher than in the control, and the addition of flour from wine lees in the amount of 20% caused a 3-fold increase in antiradical activity compared to the control sample. On the other hand, the antioxidant activity using the free radical DPPH in muffins with 10% and 20% content of flour from wine lees was 5 and 10 times higher, respectively, compared to the control. In the studies of Mala et al. [64], the antiradical activity using the free radical DPPH in cookies was 123.7 mg TX/100 g of product. The addition of 5% powdered pineapple peel to baking cookies increased antioxidant activity by 182.6% compared to the control, and the use of 10% powdered pineapple peel increased antiradical activity by three and a half times compared to the control [64]. The addition of 20% soy flour to baking muffins increased DPPH antiradical activity by 11.36% compared to the control [46].

It should therefore be stated that the type of additive will largely determine the antioxidant activity of finished products with the additive, which was also confirmed in this research.

### 2.4. The Influence of Polysaccharide Fraction Residue Derived from Unripe Apple Oliwka and Pyros Varieties on the Tocopherols and Phytosterols Content in Muffins

It should be noted that the content of alpha, gamma, and delta tocopherol was determined in the polysaccharide fraction residue from both varieties of unripe apples. In the case of muffins with a share of polysaccharide fraction residue, beta tocopherol also appears (Table 1 and Table 4). This is not a coincidence because both wheat flour and grape seed oil were used to bake the muffins, which are a source of tocopherols and tocotrienols, including beta tocopherol [65]. It was shown that the content of delta tocopherol, alpha tocopherol, and beta tocopherol was the highest in the control muffins, and by adding SO, the content of these tocopherols decreased by up to two times. An exception in this respect was gamma tocopherol because its content in the control muffin was lower than in the muffins with the participation of 10% SO (Table 4).

This is consistent with the results of other authors [65,66] because the muffin baked goods were based on wheat flour and grape seed oil, which are ingredients rich in tocopherols and phytosterols. Adding an apple polysaccharide fraction residue reduces the content of these valuable compounds. This is in line with previous studies, which reported tocopherol losses of 20 to 60% during the baking of bread and cakes [67,68]. Losses of tocopherols may be caused by several stages in the production of cookies. According to Hidalgo and Brandolini [67], the kneading stage led to degradation of tocopherol content, which can be attributed to their oxidation by oxygen from the air during kneading and thermal degradation during baking, which is, however, of lesser importance [67,68]. They achieved significantly lower tocopherol degradation than in our case, which results from the mixing time of the ingredients (2 min vs. 10 min in our study). In the studies of the above author Hildago Brandolini [67], the mixing of ingredients was performed for two minutes, resulting in smaller losses of tocopherols. A shorter time of this operation limited the losses of tocoferols during dough mixing and its aeration and thus limited the oxidation of tocopherols. This is in contrast to our studies because the losses were greater due to the longer mixing time (Table 4). Considering the results presented in the paper, both the stages of muffin production (especially baking temperature) and the level of additive will have an impact on the tocopherol content in the tested product. According to Mildner-Szkudlarz et al. [69], the loss of tocopherols decreased with increasing temperature, while the loss of tocotrienols increased with increasing baking temperature. It was found in the work of Mildner-Szkudlarz et al. [69] that muffins made with raspberry jam baked at 240 °C had approximately 27% higher alpha-tocopherol recovery than the same samples baked at 140 °C.

Considering the content of phytosterols, it should be clearly stated that their amount was varied in muffins with the addition of apple polysaccharide fraction residue. It was noted that in the control muffins, the amount of campesterol was low, while with the addition of polysaccharide fraction residue, the content of this phytosterol increased even 18-fold (Table 4). Unfortunately, cholesterol was not noted in the muffins, but its amount was noted in the apple polysaccharide fraction residue (Table 1 and Table 4). The amount of stigmasterol was the highest in the control muffin, and its content in muffins with the participation of polysaccharide fraction residue from unripe apples decreased even twofold. The content of beta sitosterol was noted only in the control, while the amount of cleosterol was at the level of 18.99 mg per 100 g of dry matter in the control muffin, and after the addition of polysaccharide fraction residue from apples, the amount of this phytosterol increased by up to 50%. This is due to the fact that the amount of beta-sitosterol is high in grape seed oil (66.6. to 67.4 mg/kg) and the amount of cleosterol is low (0.9 to 0.94 mg/kg), while the amount of campesterol was from 0.1 to 9.3 mg/kg in oil [66]. Therefore, in the control muffins, the amount of this phytosterol was much lower than in the muffins with the addition of polysaccharide fraction residues, which are rich in campesterol (Table 1 and Table 4).

Generally it should be emphasized that the grape seed oil used for baking muffins is not only a rich source of tocopherols but also phytosterols such as beta sitosterol and stigmasterol, which is why there is quite a large amount of these compounds in muffins. It can be stated that the additives from the polysaccharide fraction residue from unripe apples increased the content of cleosterol and campesterol. The additives did not have a positive effect on the remaining phytosterols. The negative effect of high temperatures of baking muffins on the content of tocopherols and phytosterols should be noted [65,69].

### 2.5. Influence of Polysaccharide Fraction Residue Derived from Unripe Apple Oliwka and Pyros Varieties on Quality Parameters of Muffins

Table 5 presents the hardness of the analyzed muffins with the addition of polysaccharide fraction residue from unripe apples. It was found that 5% and 10% addition of polysaccharide fraction residue from apples of both apple varieties did not affect the hardness of the cookies. In the study by Mala et al. [64], the addition of powdered pineapple peel to baking cookies caused a decrease in hardness from 1.82 N in the control to 1.47 N and 1.42 N, respectively, in cookies with 5% and 10% addition of powdered pineapple peel in relation to the flour used to bake the cookies. The hardness of cookies with 2% addition of powdered pomegranate peel caused a twofold increase in hardness compared to the control [70]. Comparing the results obtained in this work with the results of Male et al. [64] and Giri et al. [70], it can be concluded that the polysaccharide fraction residue from both apple varieties is a much better additive, as it does not affect the hardness of the analyzed muffins (Table 5), as was the case after using powdered pineapple or pomegranate peel.

The volume of muffins decreased after adding the polysaccharide fraction residue from apples, and the specific volume and volume per 100 g of flour were identical, i.e., did not change after using additives (Table 5). In the study by Giri et al. [70], powdered pomegranate peel was used to bake muffins in the amount of 2–10% of the amount of flour provided in the recipe. With the increase in the percentage of the additive, the volume decreased [70]. In terms of specific volume, the addition of pomegranate peel also caused a decrease in these parameters [70]. The volume of the control sample was 71.16 mL and the addition of 6% powdered apple peel to baking muffins caused it to decrease to 68.96 mL. On the other hand, the addition of powdered apple peel did not affect the specific volume of muffins [46]. Comparing the results of Giri et al. [70] and Kaur et al. [46] with the results obtained in this work, it can be concluded that the additives used in these works for baking muffins slightly reduced the volume of the final product but they did not affect the specific volume and volume per 100 g flour (Table 5).

## 3. Materials and Methods

### 3.1. Research Material

#### 3.1.1. Reagents

All reagents were of analytical grade. Analytical reagents and chemicals were purchased from Sigma Aldrich (St. Louis, MO, USA), including stable free radicals: DPPH (2,2-diphenyl-1-picrylhydrazyl) and ABTS (2,2-azino-bis (3-ethylobenzothiazoline-6-sulphonic acid)-diamonium salt).

The raw materials for the muffin production were bought from a local market, from specialized stores (Krakow, Poland).

#### 3.1.2. Materials

In the first stage of research materials were the polysaccharide fraction residues after isolation of starch from two varieties of unripe apples, Oliwka and Pyros, which were cultivated at the Experimental Farm of the University of Agriculture in Krakow, Poland (Garlica Murowana, Zielonki). Unripe apples were harvested in May 2021, and their fruits were green and did not exceed 3.5 cm in diameter and weight of 35–40 g.

In the second stage of the study, the materials were wheat muffins with 5 and 10% polysaccharide fraction residue created after starch isolation from the above-mentioned two apple varieties: Oliwka and Pyros.

#### 3.1.3. Isolation of Polysaccharide Fraction Residue from Unripe Apples

About 1 kg of unripe apples without stalks were poured with 1.5 L of distilled water and crushed with a Zelmer ZHM0861X mixer (Rzeszów, Poland). Then, 1 g of xylanase (X2753 Sigma, St. Lous, MO, USA) was added and then the whole was mixed with a mechanical mixer 80 RW 20 (IKA, Königswinter, Germany) for 2 h. Then, the sample was filtered through a 315 μm gauze, then centrifuged for 15 min at 2260× *g* (temp. 23 °C) in order to isolate the starch [29]. Then, the upper layer of the slurry (the polysaccharide fraction residue) was removed and air-dried (SF55 Memmert GmbH, Schwabach, Germany). The dry polysaccharide fraction residue was ground on a pulverisette 2 ball mill (Ftitsch, Idar-Oberstein, Germany) and named polysaccharide fraction residue from unripe apples of two varieties, Pyros and Oliwka.

#### 3.1.4. Preparation of Muffins

Table 6 shows the recipe for the muffins. The control samples consist of wheat flour type 450 (PZZ, Kraków, Poland) and baking powder (Dr. Oetker, Gdańsk, Poland), sugar (Sodzucker Polska S.A., Wrocław, Poland), salt (CIECH, Kujawy, Poland), lemon peel (Dr. Oetker, Gdańsk, Poland), and vanilla sugar (Dr. Oetker, Gdańsk, Poland). All the ingredients were mixed for 5 min in a bowl. In another bowl, eggs (Biedronka. Kraków, Poland) were mixed with yogurt (Bakoma S.A, Warszawa, Poland) and grape seed oil (Monini-Polska Sp.zoo, Poznań, Poland). The wet ingredients were poured into the loose ingredients and gently mixed for 5 min with a spoon just until the ingredients were combined. The dough was transferred to muffin cups and baked at 190 °C for approx. 20 min. A Condo 20608 oven (MIWE, Arnstein, Germany) was used for baking. In order to obtain muffins with the polysaccharide fraction residue derived from unripe apple varieties Oliwka and Pyros, part of the wheat flour type 450 was replaced in the recipe, and the muffin preparation method was the same as described above. A photo of the selected muffins is provided in the Supplementary Materials (Figure S1).

### 3.2. Methods

The following determinations were made in both the polysaccharide fraction residue samples and the muffins:

#### 3.2.1. Preparation of Extracts for Analysis of Bioactive Compounds (Polyphenols)

Extracts from the research material were obtained using 80% ethanol in a ratio of 1:50 (*v*/*v*) at a temperature of 23 °C for 2 h without access to light with continuous shaking (Memmert, WB 22, Schwabach, Germany). Then, the extracts were centrifuged (MPW-350, Warsaw, Poland) at 1050× *g* for 15 min (temp. 23 °C). The obtained extracts were stored in a freezer (−20 °C) until further analysis.

#### 3.2.2. Total Phenolic Content (TPC)

Total phenolic content (TPC) was determined by a spectrophotometric method using the Folin–Ciocalteu reagent (F-C reagent), according to Singleton et al. [71]. The absorbance was measured using Helios Gamma 100–240 (Runcorn, UK) at the wavelength λ = 760 nm. The results were expressed to mg catechin or gallic acid/100 g d.m. Measurements were performed in duplicate.

#### 3.2.3. Determination of Total Polyphenols (TPC), Phenolic Acids, and Flavonols

Determination of total polyphenols (TPC), phenolic acids, and flavonols according to Mazza et al. [72] with modification by Oomah et al. [73]. The absorbance was measured spectrophotometrically (Helios Gamma, 100–240, Runcorn, UK) at a wavelength of λ = 280 nm (TPC), λ = 320 nm (phenolic acids), λ = 360 nm (flavonols). The results were expressed as follows: TPC—mg catechin/100 g d.m.; phenolic acids—mg ferulic acid/100 g d.m.; flavonols—mg quercetin/100 g d.m. Measurements were performed in duplicate.

#### 3.2.4. Flavonoid Content Determination

Flavonoid content was determined by a spectrophotometric method according to El Hariri et al. [74]. The flavonoid content was expressed as mg rutin/100 g d.m. Measurements were performed in duplicate.

#### 3.2.5. Antioxidant/Antiradical Activities

##### Determination of Antiradical Activity Using Synthetic Radical ABTS

Antiradical activity was assessed using analytical methods, namely ABTS [75]. The bleaching rate of ABTS+• in the presence of the sample was monitored at 734 nm using Helios Gamma 100–240 (Runcorn, UK) spectrophotometer. Radical scavenging activity was measured as Trolox Equivalents Antioxidant Capacity (TEAC) (mg Trolox per g of sample’s dry mass). Trolox solutions used for the calibration curve were used in the concentration range 0–2.5 mM (r^2^ = 0.9957). Measurements were performed in duplicate.

##### Determination of Antiradical Activity Using Synthetic Radical DPPH

Determination of antiradical activity by synthetic DPPH radical according to Brand-Williams et al. [76]. Absorbance of the sample extracts was determined with a spectrophotometer at λ = 517 nm. The results were expressed in mg/g d.m. of Trolox equivalent (TEAC). Measurements were performed in duplicate.

##### Determination of Antioxidant Activity Using Molybdenum Reducing Antioxidant Power (FOMO)

The molybdenum-reducing capacity of the samples was evaluated following a modified version of the Prieto et al. [77] method using a Jenway spectrophotometer (6405 UV/VIS, Jenway Ltd., Dunmow, UK) at λ = 700 nm. Trolox was employed as the standard (concentration range: 10–1000 mg/L; r^2^ = 0.998), and results were expressed in mg/g of Trolox equivalents. Measurements were performed in duplicate. The samples were extracted with 80% ethanol at a volume to mass ratio of 40:1 (*v*/*w*) for 2 h. After centrifugation at 4000× *g* (temp. 23 °C) (Rotofix 32 A, Hettich, Germany) for 10 min, the supernatant was used for measurement.

##### Determination of Tocopherols and Phytosterols in Food by Gas Chromatography

Determination of tocopherols and phytosterols in food by gas chromatography according to Hussain et al. [78], Oracz et al. [79], and Zhang et al. [80]. Chromatographic analysis was carried out using Shimadzu GC2010Plus Chromatograph with FID detector (Shimadzu corp., Kyoto, Japan) using an SH-5MS (length 30 m; diameter 0.25 mm; film thickness 0.25 µm) column. The injector temperature was 280 °C, and the temperature program was as follows: isothermal holding at 285 °C for 5 min, followed by an increase to 290 °C at a rate of 5 °C/min and isothermal holding for 19 min. The carrier gas was nitrogen (0.92 mL/min) with a split ratio of 1:10. Phytosterols were identified by comparing their retention times with standards. Measurements were performed in duplicate.

#### 3.2.6. Quality Parameter Determination of Muffins

##### Texture Profile Analysis

The instrumental texture measurements of the muffins were made with a TA-X2 plus Texture Analyzer (Stable Microsystems, Godalming, UK) using Texture expert software (Texture Exponent version 6.1.16.0, Stable Microsystems, Godalming, UK). The muffins were placed on the HDP/BSG platform and the cutting test (biscuit cutting) was performed at a speed of 2 mm/s. Maximum cutting force was assigned as hardness. Values were the mean of at least three replicates for each formulation. Measurements were performed four times.

##### Volume Measurement

After baking and cooling (1 h at temperature 23 °C), the volume of the muffins was assessed using a Volscan Profiler 600 laser measuring device (Stable Micro Systems, Godalming, UK). Measurements were performed four times.

##### Statistical Analysis

Analysis of variance was performed (Duncan’s test) at a significance level of 0.05 using Statistica v. 13.0 software (Statsoft, Tulsa, OK, USA). All measurements were performed at least in duplicate. The results were presented as the average with standard deviation. Pearson correlation coefficient was also calculated using Excel (Microsoft Office, Microsoft Corporation, Redmond, WA, USA).

## 4. Conclusions

It was found that the polysaccharide fraction residue isolated from two unripe apple varieties, Oliwka and Pyros, contains a significant amount of polyphenols, phenolic acids, flavonols, and flavonoids, has a high antioxidant potential, and can become a valuable and attractive functional ingredient to formulate functional food.

The study demonstrates that polysaccharide fraction residue isolated from unripe apples can be used in baked goods and significantly improves antioxidant capacity without affecting hardness and volume of the final product. This residue significantly increases polyphenols, flavonoids, and phenolic acids and the content of campesterol (eighteen-fold increase) and cleosterol (50% increase) in muffins.

It was shown that the polysaccharide fraction residue from Oliwka apples was more beneficial as an addition to muffins than the residue from Pyros apples, both in terms of the physical properties of these muffins and the content of antioxidants and antioxidant capacity of the final product.

This research clearly outlines strategies for the future management of unripe fruit waste, especially in the context of our planet’s climate change, and should be continued.

## Figures and Tables

**Table 1:** Content of tocopherols and phytosterols in analyzed polysaccharide fraction residue after isolation of starch from Pyros and Oliwka unripe apples.

Sample	α-Tocopherol	β-Tocopherol	γ-Tocopherol	δ-Tocopherol	Campesterol	Cholesterol	Stigma-Sterol	Beta- Sitosterol	Cleosterol
[mg/100 g d.m.]									
SP	1.62 ± 0.1 ^B^	n.d.	3.05 ± 0.05 ^B^	0.9 ± 0.08 ^A^	100.54 ±1.34 ^A^	2.3 ± 0.2 ^A^	2.32 ± 0.3 ^A^	297 ± 0.29 ^B^	2.0 ± 0 ^A^
SO	0.78 ± 0 ^A^	n.d.	2.73 ± 0.02 ^A^	1.7 ± 0 ^B^	111.28 ± 3.27 ^B^	2.2 ± 0 ^A^	2.3 ± 0.3 ^A^	259 ± 0.72 ^A^	2.1 ± 0 ^B^

Different letters indicate statistically significant differences (Duncan’s test, significance level α ≤ 0.05); n.d.—not detected; SO—polysaccharide fraction residue from unripe apple variety Oliwka; SP—polysaccharide fraction residue from unripe apple variety Pyros.

**Table 2:** Content of polyphenols and flavonoids, phenolic acids, and flavonols in analyzed muffins with addition of polysaccharide fraction residue derived from unripe apples.

Scheme 100	Total Polyphenols with F-C Reagent (Catechin Equivalent)	Total Polyphenols Without F-C Reagent (Catechin Equivalent)	Flavonoids (Rutin Equivalent)	Phenolic Acids (Ferulic Acid Equivalent)	Flavonols (Quercetin Equivalent)
	[mg/100 g d.m.]				
K	50.15 ± 0.00 ^A^	64.86 ± 1.82 ^A^	13.39 ± 1.76 ^A^	1.34 ± 0.73 ^A^	0.88 ± 0.87 ^A^
5% MSP	67.66 ± 0.00 ^B^	75.22 ± 14.66 ^B^	23.82 ± 0.49 ^B^	3.74 ± 0.72 ^B^	1.68 ± 0.28 ^B^
10% MSP	68.75 ± 0.09 ^C^	82.13 ± 3.45 ^C^	26.63 ± 1.69 ^C^	4.89 ± 0.28 ^C^	3.24 ± 0.41 ^C^
5% MSO	71.31 ± 0.63 ^D^	87.31 ± 1.17 ^D^	27.2 ± 2.43 ^C^	5.17 ± 0.32 ^D^	1.74 ± 0.77 ^B^
10% MSO	92.09 ± 0.63 ^E^	118.40 ± 18.17 ^E^	30.86 ± 1.14 ^C^	6.67 ± 0.28 ^E^	3.24 ± 0.96 ^C^

Different letters indicate statistically significant differences (Duncan’s test, significance level α ≤ 0.05); Abbreviations: K—control muffins; 5% MSP—muffins with 5% share of polysaccharide fraction residue from Pyros variety unripe apples; 10% MSP—muffins with 10% share of polysaccharide fraction residue from Pyros variety unripe apples; 5% MSO—muffins with 5% share of polysaccharide fraction residue from Oliwka variety unripe apples; 10% MSO—muffins with 10% share of polysaccharide fraction residue from Oliwka variety unripe apples.

**Table 3:** Antiradical and antioxidant activities in analyzed muffins with addition of polysaccharide fraction residue derived from unripe apples.

Sample	ABTS [mg TX/g d.m.]	DPPH [mg TX/g d.m.]	FOMO [mg TX/g d.m.]
K	4.95 ± 0.19 ^A^	1.99 ± 0.00 ^A^	10.82 ± 1.12 ^A^
5% MSP	6.35 ± 0.23 ^B^	2.01 ± 0.10 ^A^	57.86 ± 059 ^B^
10% MSP	7.17 ± 0.00 ^C^	2.09 ± 0.15 ^A^	107.03 ± 0.80 ^D^
5% MSO	6.18 ± 0.08 ^B^	2.21 ± 0.00 ^B^	80.45 ± 1.10 ^C^
10% MSO	7.55 ± 0.07 ^C^	2.02 ± 0.07 ^A^	141.66 ± 1.52 ^E^

Different letters indicate statistically significant differences (Duncan’s test, significance level α ≤ 0.05).

**Table 4:** Tocopherols and phytosterols in selected samples of muffins with addition of polysaccharide fraction residue derived from unripe apples.

Sample	Tocopherols			
	[mg/100 g d.m.]			
	α-tocopherol	β-tocopherol	γ-tocopherol	δ-tocopherol
K	34.44 ± 0.28 ^C,^*	5.99 ± 0.14 ^C^	2.29 ± 0.1 ^A^	3.57 ± 0.23 ^C^
5% MSO	19.87 ± 0.89 ^A^	2.15 ± 0.31 ^A^	2.4 ± 0.15 ^A^	0.97 ± 0.11 ^A^
10% MSO	20.58 ± 1.21 ^B^	3.3 ± 0.57 ^B^	3.89 ± 0.13 ^B^	1.89 ± 0.13 ^B^
	Phytosterols			
	[mg/100 g d.m.]			
	Campesterol	Stigmasterol	β-sitosterol	Cleosterol
K	0.79 ± 0.15 ^A^	1.35 ± 0.04 ^B^	0.65 ± 0.2 ^A^	18.99 ± 0.11 ^A^
5% MSO	16.88 ± 0.21 ^C^	0.64 ± 0.25 ^A^	-	30.82 ± 0.53 ^C^
10% MSO	9.24 ± 0.34 ^B^	0.86 ± 0.17 ^A^	-	21.53 ± 0.74 ^B^

* Different letters indicate statistically significant differences (Duncan’s test, significance level α ≤ 0.05).

**Table 5:** Physical parameters of analyzed muffins with addition of polysaccharide fraction residue derived from unripe apples.

Sample	Hardness [N]	Volume [mL]	Specific Volume [mL/g]	Volume per 100 g Flour [mL]
K	2.18 ± 0.25 ^A^	71.37 ± 1.31 ^C^	1.85 ± 0.05 ^A^	142.73 ± 2.63 ^A^
5% MSP	2.49 ± 0.36 ^A^	70.88 ± 1.52 ^C^	1.82 ± 0.01 ^A^	141.77 ± 3.04 ^A^
10% MSP	2.37 ± 0.29 ^A^	63.06 ± 5.48 ^B^	1.80 ± 0.03 ^A^	126.11 ± 10.95 ^A^
5% MSP	2.36 ± 0.12 ^A^	68.68 ± 1.61 ^B^	1.84 ± 0.01 ^A^	137.36 ± 3.22 ^A^
10% MSO	2.34 ± 0.04 ^A^	59.96 ± 3.22 ^A^	1.75 ± 0.07 ^A^	119.92 ± 6.44 ^A^

Different letters indicate statistically significant differences (Duncan’s test, significance level α ≤ 0.05).

**Table 6:** Recipe for baking muffins in laboratory conditions.

Ingredients:	Standard	5% Polysaccharide Fraction Residue	10% Polysaccharide Fraction Residue
	[g]		
Flour	150	142.5	135
Baking powder	5	5	5
Crystal sugar	75	75	75
Salt	5	5	5
Lemon peel	13.5	13.5	13.5
Vanilla sugar	8	8	8
Natural yogurt	125	125	125
Whole egg	1	1	1
Grape seed oil	62.5	62.5	62.5
Apple polysaccharides fraction residue	-	7.5	15

## Data Availability

Data are contained within the article.

## Supplementary Materials

The following supporting information can be downloaded at: https://www.mdpi.com/article/10.3390/molecules30102189/s1. Figure S1: The prepared muffins. From the left: control, 5% MSO—muffins with 5% share of polysaccharide fraction residue from Oliwka variety unripe apples; 10% MSO—muffins with 10% share of polysaccharide fraction residue from Oliwka variety unripe apples. Figure S2: Polyphenol and flavonoid content and antioxidant potential of muffins with polysaccharide fraction residue.

## Abbreviations

The following abbreviations are used in this manuscript:

K	control muffins
5% MSP	muffins with 5% share of polysaccharide fraction residue from Pyros variety of unripe apples
10% MSP	muffins with 10% share of polysaccharide fraction residue from Pyros variety of unripe apples
5% MSO	muffins with 5% share of polysaccharide fraction residue from Oliwka variety of unripe apples
10% MSO	muffins with 10% share of polysaccharide fraction residue from Oliwka variety of unripe apples
TPC	Total phenolic content
DPPH	2,2-diphenyl-1-picrylhydrazyl
ABTS	2,2-azino-bis(3-ethylobenzothiazoline-6-sulphonic acid-diamonium salt
FOMO	Molybdenum Reducing Antioxidant Power
SO	polysaccharide fraction residue from Oliwka unripe apples
SP	polysaccharide fraction residue from Pyros unripe apples
