# Supplementary material for: Quality and Content of Bioactive Compounds in Muffins with Residue After Isolation of Starch from Unripe Apples (Malus domestica Borkh)
Source: Molecules. 2025 May 16;30(10):2189. doi: 10.3390/molecules30102189 (PMC12114245; doi:10.3390/molecules30102189)
Supplement: Supplementary file 1 [file molecules-30-02189-s001.zip › molecules-3604106-supplementary.pdf]

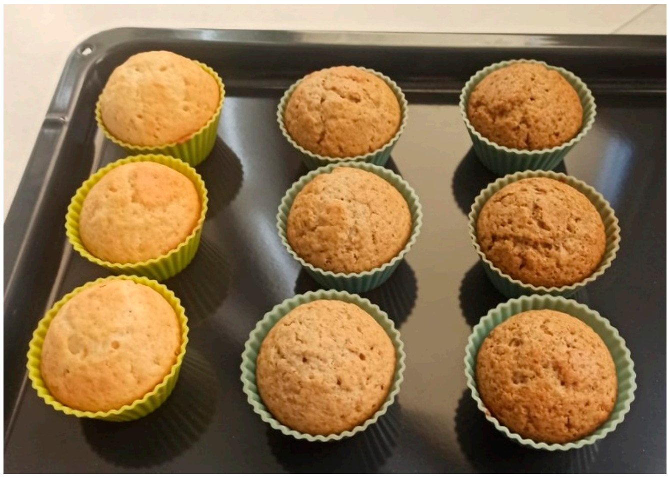

Figure S1. From the left: control, 5% MSO – muffins with 5% share of polysaccharide fraction residue from Oliwka variety apples; 10% MSO – muffins with 10% share of polysaccharide fraction residue from Oliwka variety apples

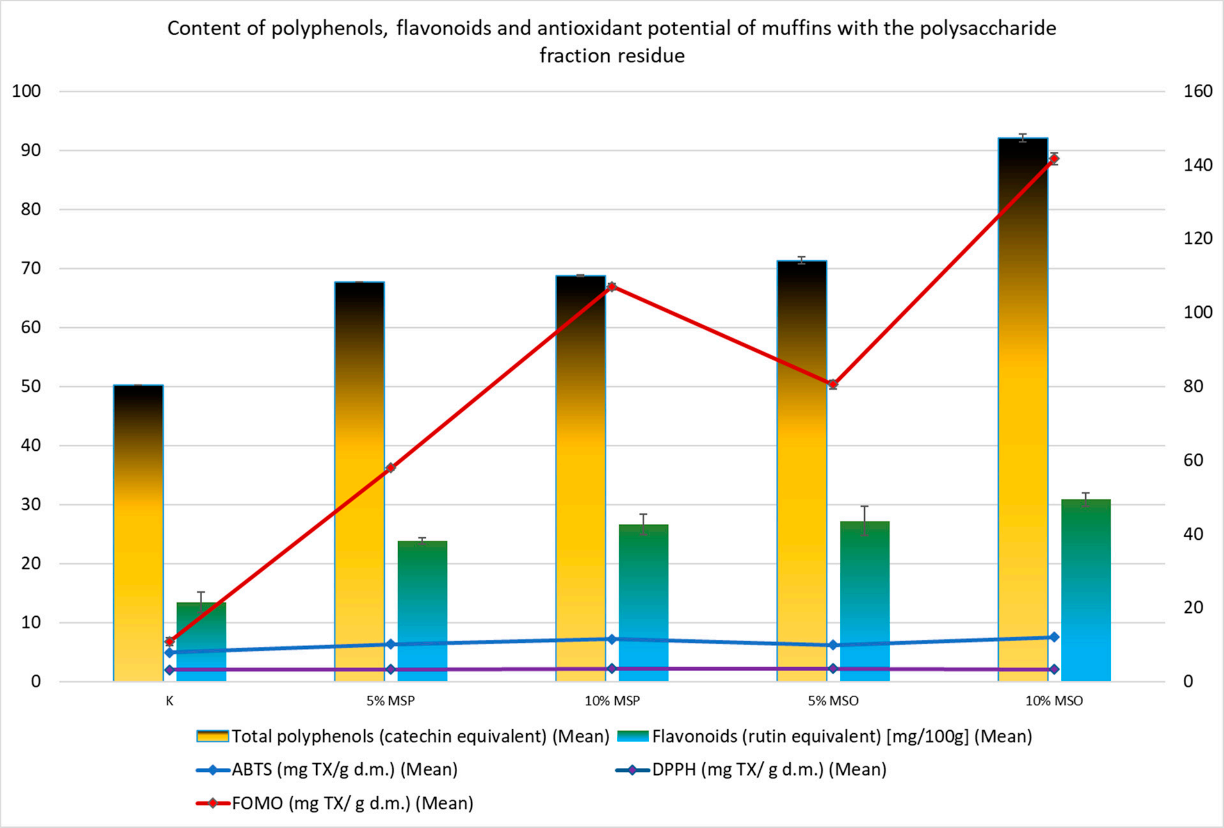

Figure S2. Polyphenol and flavonoid content and antioxidant potential of muffins with polysaccharide fraction residue
